# Comparison of Percutaneous Radiofrequency Ablation for Subcapsular and Non-Subcapsular Colorectal Cancer Liver Metastases

**DOI:** 10.3389/fonc.2021.678490

**Published:** 2021-05-14

**Authors:** Hongjie Fan, Xiaoyan Wang, Jiali Qu, Wei Lu, Shufeng Xu, Xia Wu, Jingya Xia, Yanhua Zhang, Jihong Sun, Xiaoming Yang

**Affiliations:** ^1^ Department of Radiology, Sir Run Run Shaw Hospital, School of Medicine, Zhejiang University, Hangzhou, China; ^2^ Department of Pathology, Sir Run Run Shaw Hospital, School of Medicine, Zhejiang University, Hangzhou, China; ^3^ Image-Guided Bio-Molecular Intervention Research, Department of Radiology, University of Washington School of Medicine, Seattle, WA, United States

**Keywords:** colorectal cancer liver metastases, radiofrequency ablation, local tumor progression, minimal ablative margin, complications

## Abstract

**Purpose:**

To evaluate the efficacy and safety of percutaneous radiofrequency ablation (RFA) for subcapsular colorectal cancer liver metastases (CLMs).

**Materials and Methods:**

With the approval of the Institutional Review Board, the clinical data of CLM patients who underwent percutaneous RFA for the first time from August 2010 to August 2020 were continuously collected. All CLMs were divided into subcapsular and non-capsular groups. Baseline characteristic data, technical effectiveness, minimal ablative margin, complications, local tumor progression (LTP), and overall survival (OS) between the two groups were analyzed using the t-test or chi-square test. A Cox regression model was used to evaluate the prognostic factors of LTP.

**Results:**

One hundred and ninety-nine patients (124 males; mean age, 60.2 years) with 402 CLMs (221 subcapsular; mean size, 16.0 mm) were enrolled in the study. Technical effectiveness was achieved in 93.5% (376/402) of CLMs, with a major complication rate of 5.5%. Compared with non-subcapsular tumors, the minimal ablative margin achieved in subcapsular CLM was smaller (χ^2^ = -8.047, P < 0.001). With a median follow-up time of 23 months (range, 3−96 months), 37.1% of the tumors had LTP. The estimated cumulative OS at 1, 3, and 5 years was 96.1%, 66.0%, and 44.2%, respectively. There were no statistically significant differences between the two groups in terms of technical effectiveness (χ^2^ = 0.484, P = 0.487), major complications (χ^2^ = 0.082, P = 0.775), local tumor progression-free survival (LTPFS) (χ^2^ = 0.881, P = 0.348), and OS (χ^2^ = 2.874, P = 0.090). Minimal ablative margin, tumor size (≥20 mm), and technical effectiveness were predictors of LTP (all P < 0.05).

**Conclusion:**

RFA is a safe and effective technique for local tumor control of subcapsular CLMs.

## Introduction

Colorectal cancer (CRC) is the third most common cause of cancer death in the United States (1), and more than 25% of patients develop liver metastases during the course of the disease (2). Although surgical treatment of colorectal cancer liver metastases (CLMs) has made great progress, surgical resection is only suitable for less than 20% of affected patients (3). Percutaneous radiofrequency ablation (RFA) has been widely used in the comprehensive treatment of CLMs and has achieved satisfactory results in local tumor control and overall survival (OS) with good safety (4).

Nevertheless, the treatment of subcapsular tumors (primary hepatocellular carcinoma or CLMs) remains controversial. Traditionally, RFA, the most common percutaneous local thermal ablation technique, has not been completely suitable for the treatment of subcapsular tumors. This may be due to the limited treatment window and the tendency to be affected by structures, such as ribs and diaphragms, resulting in technical difficulties in electrode placement and an increased risk of major complications such as tumor seeding (5, 6), bleeding, and thermal damage to adjacent crucial structures (such as the liver dome, abdominal wall, gallbladder, and intestine) (7–9). Therefore, the location of the subcapsular tumor is still considered a relative contraindication for thermal ablation (7).

To the best of our knowledge, there are conflicting results in multiple studies on the safety and efficacy of RFA for subcapsular tumors. Recently, Han et al. showed that subcapsular tumor location was an important independent risk factor (hazard ratio [HR], 1.9; 95% confidence interval [CI]: 1.1−3.1) for local tumor progression–free survival (LTPFS) of CLMs (10). Previous studies have also reported similar results (6, 11–13). The difference is that some studies have shown no statistically significant difference in technical success, major complications (neoplastic seeding or bleeding), and clinical effects (local tumor progression [LTP], or OS) in the treatment of subcapsular and non-subcapsular tumors with RFA (8, 13–16). They believe that when appropriate and optimized assistive technologies are available, the RFA effect of subcapsular tumors can be comparable to that of non-subcapsular tumors (7, 15). Teratani et al. (17) evaluated the efficacy and safety of RFA for tumors in high-risk locations, and the results showed no statistically significant difference in early complications and LTP after RFA in high-risk locations compared to normal locations.

To further clarify these issues, we retrospectively compared the complications, technical effectiveness, intrahepatic distribution of CLMs, and mid-to-long-term treatment effects (LTP and OS) of percutaneous RFA for subcapsular and non-subcapsular CLMs and analyzed the predictors of LTP.

## Materials and Methods

This retrospective study was approved by the Institutional Review Board of Sir Run Run Shaw Hospital, Zhejiang University School of Medicine, and written informed consent was obtained from all patients before the RFA procedure.

### Patients

We continuously collected clinical data of CLM patients who underwent percutaneous RFA for the first time between August 2010 and August 2020 in our hospital. For patients eligible for RFA treatment, two to three physicians with more than 10 years of RFA treatment experience completed this procedure. Before RFA, the primary tumors of all patients were treated by surgery. Additionally, patients were treated with comprehensive management strategies as appropriate for primary tumor control, such as chemotherapy, stereotactic body radiation therapy (SBRT), targeted therapy, and immunotherapy. The number of CLMs > 5, the size of CLMs > 5 mm, uncorrectable coagulation dysfunction, evidence of vascular invasion, Child-Pugh grade C liver function, vital organ failure, sepsis, and biliary tract infections were considered as the exclusion criteria for RFA treatment. In the process of data collection, cases were excluded under the following circumstances (1): Incomplete diagnosis and treatment records (such as progress notes, surgical records, and laboratory examinations) (2); lack of periprocedural imaging (CT or MRI); and (3) less than 3 months of follow-up time.

### RFA Procedure

All patients underwent dynamic contrast-enhanced (DCE) CT (GE LightSpeed VCT, Boston, Massachusetts, USA) or MRI (GE Signa HDx 3.0T, Boston, Massachusetts, USA) and laboratory tests (including liver function, blood routine, tumor markers, etc.) before RFA. The RFA procedure was performed under general anesthesia. Continuous electrophysiological monitoring was performed by a team of professional anesthesiologists. The RF-3000 (Boston Scientific, Boston, USA) instrument was used for percutaneous intrahepatic RFA for tumor treatment under the guidance and monitoring of CT (Siemens Definition AS, Erlangen, Germany) as needed. Based on the size and location of the tumor, we selected the appropriate needle or needle cluster and a radiofrequency electrode and determined the needle entry point and angle under CT guidance. RFA was performed after the front end of the electrode reached the edge of the lesion and satisfactory coverage of the tumor by the sub-electrode was confirmed by CT scan. An output power of 100–150 W was selected according to the actual situation, and controlled by the feedback of the load impedance, with a treatment time of 5–15 min. The area of the expected ablation defect that the RFA was intended to achieve exceeded the tumor margin by at least 5 mm. Finally, the electrode path was cauterized when the needle was retired to prevent neoplastic seeding or bleeding. The percutaneous saline isolation method was used to create operable conditions when the procedure was affected by organs such as the stomach and intestines. DCE CT or MR was immediately performed after RFA to evaluate the ablation defect zone and surrounding margin within 10 mm, and complications (e.g., pleural effusion and bleeding) were detected and managed immediately.

### Definition

**Figure 1:**
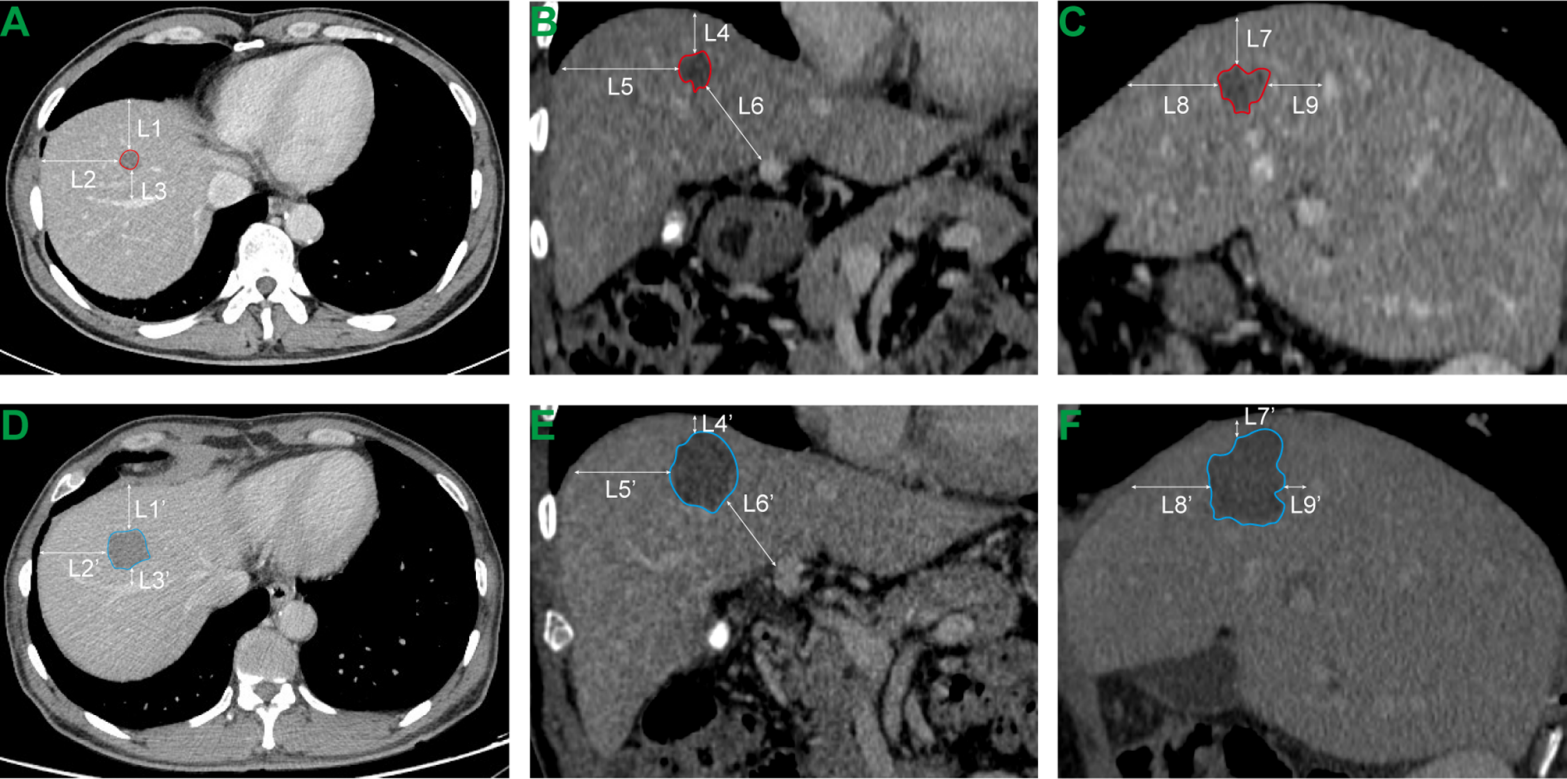
Measurement of minimal ablative margin. **(A–C)** Transverse, coronal and sagittal CT images before RFA. L1, L2,…, L9 respectively represent the distance from the tumor boundary to the selected anatomical landmark point. **(D–F)** Transverse, coronal and sagittal CT images of the same plane after RFA. L1’, L2’,…, L9’ respectively represent the distance from the perfusion defect zone generated by thermal ablation to the selected anatomical landmark point. The minimal ablative margin of RFA procedure is the minimum value of (L1-L1’), (L2-L2’),…, (L8-L8’) and (L9-L9’).

Subcapsular CLM was defined as a tumor less than 5 mm from the hepatic capsule (10). Perivascular tumor location referred to the tumor contacting the first- or second-degree branches of the intrahepatic vein with a diameter of ≥5 mm. Tumors adjacent to organs indicate that the minimum distance between the tumor and the extrahepatic organs (e.g., gallbladder, diaphragm, stomach, intestine, and kidney) is less than 5 mm (17). According to the Society of Interventional Radiology (SIR) classification system, complications can be divided into major (require therapy, < 48 hours’ minor hospitalization; require major therapy, unplanned increase in the level of care, prolong hospitalization; permanent adverse sequelae; death) and minor (no therapy or nominal therapy) complications by the outcome (18). To obtain the minimum ablation margin, we adopted the following steps (**Figure 1**): first, the anatomical landmarks around the tumor with relatively fixed positions before and after the RFA procedure were accurately selected, such as the liver capsule and secondary blood vessel branches; second, the shortest distance from the tumor to the anatomical landmark point before RFA and the ablation defect zone to the anatomical landmark point after RFA were measured; and finally, the difference between the corresponding distances measured before and after the RFA was calculated, and the smallest difference was selected as the minimal ablative margin. The entire measurement process was performed by two radiologists with more than 10 years of experience in radiological diagnosis using a previously reported method (3, 10, 19).

### Follow-Up

All patients underwent DCE CT or MR examination within 4-8 weeks after RFA as a baseline scan for future comparisons. Based on these examination methods, technical effectiveness was achieved when no evidence of residual tumor at the ablation defect zone and periphery within 10 mm was observed; otherwise, it was regarded as a technical failure. Follow-up was performed every 3 months for the first two years and every 6 months thereafter. For technical ineffectiveness and LTP or new metastases found in the liver during follow-up, RFA retreatment or other treatment strategies (such as stereotactic body radiotherapy, transcatheter arterial chemoembolization, and surgical resection) were considered based on the actual situation.

### Statistical Analysis

For the two groups of subcapsular and non-capsular tumors (patients with or without subcapsular tumor), continuous data (e.g., age, body mass index [BMI], and tumor size) were compared using Student’s t-test after the normality test and homogeneity test of variance. Counting variables and rates were analyzed and compared using the chi-square test, including baseline data characteristics of patients or tumors, technical effectiveness, complications, and LTP. In the analysis of the occurrence of LTP based on univariate analysis, variables with P less than 0.2 were included in the multivariate Cox regression model to evaluate the independent predictors of LTP. Since some studies defined the subcapsular tumor as a lesion located within 10 mm from the liver edge (9), in the subsequent subgroup analysis, we divided all tumors into four groups based on the distance (d) between the CLM and the liver edge: Group A, surface contact group (d = 0 mm); Group B, 0 mm < d ≤ 5 mm; Group C, 5 mm < d ≤ 10 mm; Group D, d >10 mm. We further used one-way analysis of variance (ANOVA) and chi-square tests to analyze the statistical differences of the above-mentioned variables in the four groups of tumors. The least significant difference method and the Student-Newman-Keuls method were used for *post hoc* tests. SPSS (version 24.0; IBM, Chicago, USA) was used for statistical analysis. Statistical significance was set at P < 0.05.

## Results

One hundred and ninety-nine patients (male, 124, 62.3%) with 494 tumors (mean, 2.5 ± 1.3, range from 1 to 5) were included in this study, with a mean age of 60.2 ± 11.3 years (range, 27−89 years). Eighty-two patients (41.2%) were associated with extrahepatic metastasis, 60 patients (30.2%) had undergone pre-RFA hepatectomy, 171 patients (85.9%) had preoperative chemotherapy, and 112 patients (56.3%) had comorbidities such as hypertension and diabetes. The most common primary tumor was of the rectum (32.8%), and the degree of differentiation was mainly medium differentiation (31.8%). The results of the pre-procedural laboratory test showed that the patients with CEA >30 ng/mL were 24.1% (48 cases) of patients with CA199 >37 U/mL and 26.1% (52 cases), respectively.

A total of 402 CLMs were ablated in this study (mean size, 16.0 ± 7.5 mm; subcapsular tumor, n = 221). Due to the limited accessibility of the treatment window, 92 unablated tumors were additionally treated with surgery, TACE, brachytherapy, and stereotactic body radiotherapy. The average treatment time and treatment power were 13.8 ± 7.8 min and 137.4 ± 36.9 W, respectively, with technical effectiveness achieved in 376 CLMs (93.5%). The median total hospital stay and post-procedural hospital stay were 11 and 4 days, respectively. There was no statistically significant difference in the baseline data of patients or tumors between the subcapsular and non-subcapsular groups (**Table 1**).

**Table 1:** Analysis of baseline characteristics of patients and tumors.

Variables	Patients without subcapsular tumor	Patients with subcapsular tumor	t/χ^2^	P
No. of patients*	62(31.2)	137(68.8)		
Sex (male)*	41(66.1)	83(60.6)	0.559	0.455
Age (y)*	61.6 ± 11.6	60.8 ± 11.3	0.459	0.647
BMI (kg/m^2^)*	23.2 ± 2.8	22.9 ± 2.9	0.588	0.557
Comorbidities*	39(62.9)	73(53.3)	1.605	0.205
Extrahepatic metastasis*	23(37.1)	59(43.1)	0.628	0.428
	Nonsubcapsular tumor	Subcapsular tumor		
No. of tumors	181	221		
Primary tumor location			6.535	0.163
Rectum	57(31.5)	75(33.9)		
Sigmoid colon	51(28.2)	73(33.0)		
Descending colon	36(19.9)	24(10.9)		
Transverse colon	8(4.4)	10(4.5)		
Ascending colon	29(16.0)	39(17.6)		
Differentiation (missing, n=66)			3.452	0.485
Low	15(9.7)	20(11.0)		
Low - medium	13(8.4)	23(12.6)		
Medium	57(37.0)	71(39.0)		
Medium - high	55(35.7)	50(27.5)		
High	14(9.1)	18(9.9)		
Synchronous metastases	96(53.0)	127(57.5)	0.790	0.374
Previous liver resection	46(25.4)	71(32.1)	2.173	0.140
Prior chemotherapy	155(85.6)	197(89.1)	1.122	0.289
Primary tumor invasion (missing, n=15)			0.210	0.647
T1-3	98(56.3)	115(54.0)		
T4	76(43.7)	98(46.0)		
CEA (ng/ml) (missing, n=4)			0.015	0.903
≤30	138(76.7)	166(76.1)		
>30	42(23.3)	52(23.9)		
CA199 (u/ml) (missing=10)			0.435	0.510
Normal (0~37)	126(71.6)	148(68.5)		
Abnormal (>37)	50(28.4)	68(31.5)		
Primary tumor lymph node metastasis	94(51.9)	104(47.1)	0.946	0.331
Minimal ablative margin (mm)	5.7 ± 2.0	3.9 ± 2.5	-8.047	<0.001
Technical effectiveness	171(94.5)	205(92.8)	0.484	0.487
Tumor size (mm)	15.3 ± 7.2	16.5 ± 7.6	1.623	0.105
Liver segment			15.076	0.035
I	0(0)	2(0.9)		
II	9(5)	18(8.1)		
III	8(4.4)	10(4.5)		
IV	27(14.9)	25(11.3)		
V	22(12.2)	15(6.8)		
VI	33(18.2)	68(30.8)		
VII	40(22.1)	38(17.2)		
VIII	42(23.2)	45(20.4)		
Perivascular tumor	44(24.3)	23(10.4)	13.847	<0.001

*Patients are divided into groups with or without subcapsular tumors for comparison. Counting data are expressed as n (%), and Pearson’s chi-square test for data analysis. Measurement data are expressed as mean ± standard deviation, and t-test for data analysis. BMI, body mass index; CEA, carcinoembryonic antigen; CA199, Carbohydrate antigen199.

### Intrahepatic Distribution of CLMs

The most common hepatic segment distribution of subcapsular CLMs was in segment VI (30.8%), followed by segment VIII (20.4%); non-subcapsular tumors were mostly distributed in segments VIII (23.2%) and VII (22.1%). The difference in the hepatic segment distribution of subcapsular and non-subcapsular tumors was statistically significant (χ^2^ = 15.076, P = 0.035). In all subcapsular tumors, the percentages adjacent to the diaphragm, gallbladder, gastrointestinal tract, and right kidney were 59.3% (131 of 221), 8.6% (19 of 221), 20.8% (46 of 221), and 12.2% (27 of 221), respectively. There was no statistically significant difference in tumor size (P = 0.105) between the two groups (**Table 1**).

### Complications

In this study, the incidence of major complications was 5.5% (11/199), including pleural effusion (5 cases), liver function impairment (2 cases), pneumothorax (2 cases), hemoperitoneum (1 case), and skin burn (1 case). The incidence of minor complications was 19.1% (38/199), with pain being the most common (26 cases). There was no statistically significant difference in the incidence of major (χ^2^ = 0.082, P = 0.775) and minor complications (χ^2^ = 1.222, P = 0.269) between patients with and without subcapsular tumors.

### Follow-Up Findings

**Figure 2:**
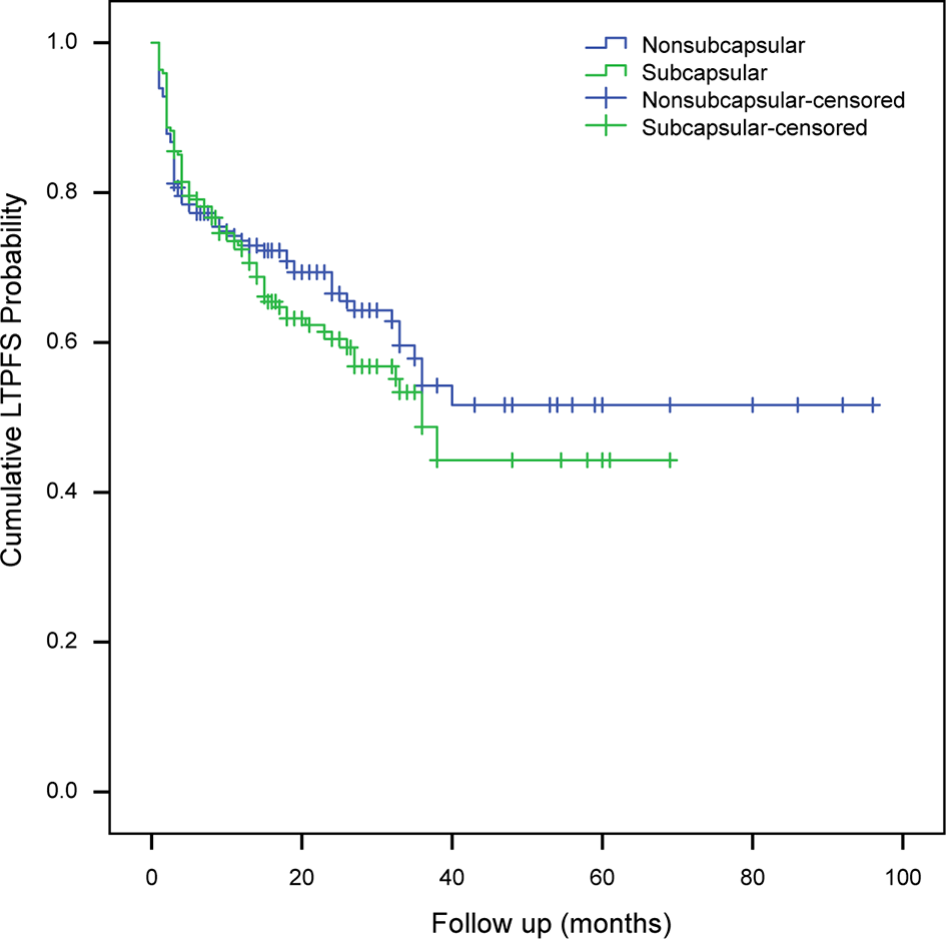
Analysis of local tumor progression-free survival of subcapsular tumors and non-subcapsular tumors. LTPFS, local tumor progression-free survival.

Compared to subcapsular tumors, non-subcapsular tumors with an average minimal ablative margin of 5.7 ± 2.0 mm obtained a more sufficient ablation margin (t = -8.047, P < 0.001). However, there was no statistically significant difference in the technical effectiveness between the two groups (χ^2^ = 0.484, P = 0.487). With a median follow-up time of 23 months (range, 3–96 months), LTP occurred in 149 of 402 (37.1%) tumors, and the first (71.1%, 106/149) and second (88.6%, 132/149) years after RFA were high incidence periods. For LTP cases, RFA retreatment (73 cases), transcatheter arterial chemoembolization (9 cases), radioactive seed implantation (5 cases), stereotactic body radiotherapy (4 cases), surgical resection (2 cases), and other comprehensive treatments were performed to control tumor progression. LTP was observed in 35.9% (65/181) of non-subcapsular tumors and 38% (84/221) of subcapsular tumors, and the difference was not statistically significant (χ^2^ = 0.188, P = 0.665). The mean LTPFS in the subcapsular tumor and non-subcapsular tumor groups estimated by the Kaplan-Meier method was 39.2 ± 2.7 months (95%CI: 33.961−44.447) and 57.0 ± 3.9 months (95%CI: 49.249−64.719), respectively. The log-rank test showed that the difference in LTPFS between the two groups was not statistically significant (χ^2^ = 0.881, P = 0.348) (**Figure 2**).

During follow-up, we observed disease-related deaths in 40 patients (20.1%). The cumulative proportion of surviving patients at 1, 3, and 5 years was 96.1%, 66.0%, and 44.2%, respectively. The estimated median survival times were 46 and 60 months in 137 patients with subcapsular tumors and 62 patients without subcapsular tumors, respectively; the difference was not statistically significant (χ^2^ = 2.874; P = 0.090).

### Subgroup Analysis

According to the distance between the tumor and the liver edge, 402 CLMs were divided into 156 in groups A, 65 in group B, 46 in group C, and 135 in group D, respectively. We compared the baseline data characteristics and follow-up data of the four groups of tumors and found that their differences in sex, CA199, location adjacent to the diaphragm, and perivascular tumor location were statistically significant (P < 0.05). Male patients were less common in group B (χ^2^ = 10.270, P = 0.016). CA199 abnormalities (>37 U/mL) were more common in groups A and D (χ^2^ = 0.018, P = 0.018). The tumors in the surface contact group (group A) were more adjacent to the diaphragm (χ^2^ = 162.186, P < 0.001), while more tumors in the D group were located perivascularly (χ^2^ = 19.574, P < 0.001). We used the Kaplan-Meier method to analyze the LTPFS of the four groups of tumors. The estimated mean LTPFS time was the longest in group C (59.5 ± 6.3 months), but the difference between the tumors in the four groups was not statistically significant (χ^2^ = 3.403, P = 0.334).

### Predictive Factors for LTP

The results of the univariate analysis are presented in **Table 2**. In univariate analysis, the differences in differentiation (χ^2^ = 12.324, P = 0.015), tumor size (χ^2^ = 30.881, P < 0.001), hepatic segment (χ^2^ = 16.348, P = 0.022), technical ineffectiveness (χ^2^ = 15.455, P < 0.001), and minimal ablative margin (t = 3.986, P < 0.001) between the LTP and non-LTP groups were statistically significant. The potential predictors obtained above together with the variables including comorbidities (χ^2^ = 2.742, P = 0.098), perivascular location (χ^2^ = 2.920, P = 0.087), and age (t = 1.843, P = 0.066) were included in the multivariate Cox regression model for further analysis. The sufficient ablative margin (OR, 0.864; 95% CI: 0.797−0.936, P < 0.001) was an independent protective factor for LTP. Tumor size ≥ 20 mm (OR = 1.894, 95% CI: 1.256−2.856, P = 0.002) and technical ineffectiveness (OR = 1.974, 95% CI:1.099−3.544, P = 0.023) were independent risk factors. The results of the multivariate Cox regression analysis of LTP are shown in **Table 2**.

**Table 2:** Univariate and multivariate Cox regression analysis for evaluating predictors of local tumor progression after radiofrequency ablation.

	Univariate analysis				Multivariate analysis		
	Non-LTP (n=253)	LTP (n=149)	t/χ^2^	P	B	OR (95%CI)	P
Comorbidities	143(56.5)	71(48.0)	2.742	0.098			
Age (y)	61.0 ± 11.4	58.9 ± 11.1	1.843	0.066			
Subcapsular	137(54.2)	84(56.4)	0.188	0.665			
Size (≥20mm)	43(17.0)	63(42.3)	30.811	<0.001	0.639	1.894(1.256-2.856)	0.002
Minimal ablative margin (mm)	5.1 ± 2.3	4.1 ± 2.5	-3.986	<0.001	-0.147	0.864(0.797-0.936)	<0.001
CEA(>30ng/ml)	57(22.7)	37(25.2)	0.311	0.577			
Perivascular tumor	36(14.2)	31(20.8)	2.920	0.087			
Technical ineffectiveness	7(2.8)	19(12.8)	15.455	<0.001	0.680	1.974(1.099-3.544)	0.023

CEA, carcinoembryonic antigen; LTP, local tumor progression; B, β regression coefficient; OR, odds ratio; CI, confidence interval.

## Discussion

In this study, approximately 55% (221/402) of CLMs were subcapsular tumors. In the univariate analysis, VI and VIII were the most common liver segments. Most subcapsular tumors are far away from the first- or second-degree branches of the intrahepatic vein (portal vein, hepatic vein) with a diameter of ≥5 mm; therefore, the heat sink effect of the vein may not be a key factor affecting the therapeutic effect of subcapsular tumors. We found that the incidence of major complications related to RFA was 5.5%, with pleural effusion being the most common, and no cases of neoplastic seeding were found. The fact that there was no statistically significant difference between major and minor complications between subcapsular tumors and non-subcapsular tumors indicates that RFA treatment for subcapsular tumors is safe. As far as we know, several past studies comparing the mid-to-long-term effects of RFA for subcapsular tumors and non-subcapsular tumors were mostly aimed at hepatocellular carcinoma (7–9, 14–16). This study is the first to systematically compare and evaluate the minimal ablative margin, technical effectiveness, complications, LTPFS and OS of subcapsular CLMs and non-capsular CLMs, which has important and unique clinical value.

The average minimal ablative margin achieved by 221 subcapsular tumors was 3.9 ± 2.5 mm, and sufficient ablation margin was more difficult to obtain in subcapsular CLMs. Generally, the smaller the minimal ablative margin achieved, the more likely the incomplete RFA of the target tumor. After thermal stimulation, the microenvironment of the residual tumor changes drastically, which may promote the accelerated growth of micro-metastases and distant tumors, resulting in faster new liver metastasis and local tumor recurrence (20–24). We confirmed that the minimal ablative margin was an important key predictor of LTP, and a sufficient minimal ablative margin was beneficial in preventing LTP. Although there was a significant statistical difference between subcapsular tumors and non-capsular tumors in the minimal ablative margin, this did not affect the technical effectiveness of the treatment and the mid-to-long-term treatment effect (LTP). Residual tumor after RFA (technical inefficiency) is also related to many other factors, such as radiofrequency energy output, the histological characteristics of the tumor. Studies believed that LTP was affected by many factors, such as perivascular tumor location, tumor size, CEA level (>30 ng/ml), node-positive primary tumor, and disease-free interval from primary resection to the diagnosis of liver metastasis (3, 10). Therefore, the occurrence of LTP should not be attributed only to the minimal ablative margin. Our results also showed that during RFA, subcapsular tumors were not as frequently affected by the heat sink effect as non-subcapsular tumors (23/221 *vs.* 44/181, χ^2 =^ 13.847, P<0.001). Besides, we used artificial ascites assisted technology to treat some technically difficult subcapsular tumors. This may be useful in preventing LTP of subcapsular tumors after RFA. These variables may potentially affect the mid-to-long-term treatment effect of RFA. The metastatic tumors included in this study were treated with RFA for the first time. Our research results indicated that tumors that have not achieved technical effectiveness for the first RFA procedure may be prone to local tumor recurrence. Although remedial RFA was useful and recommended in many studies, the technical effectiveness of the earliest RFA on tumors may be beneficial to local tumor control.

With a follow-up period of 3−96 months (median, 23 months), the total LTP rate in this study was 37.1%, and the 1-year, 3-year, and 5-year LTP rates were 26.4%, 36.4%, and 37.1%, respectively, which were lower than those reported by Shady et al. (3). The median LTPFS in this study estimated by the Kaplan-Meier method was 38 months, which is higher than the 26 months reported by Shady et al. (3). There was no significant difference (P = 0.348) between LTPFS in subcapsular tumors (39 months) and non-subcapsular tumors (57 months). From the results of the subgroup analysis, we believe that the contact of the tumor with the liver surface and even the shortest distance from the tumor to the edge of the liver are not key factors that affect the technical effectiveness and clinical efficacy of RFA.

Therefore, we believe that subcapsular metastases can be controlled by RFA and should not be regarded as a contraindication. This is consistent with the findings of previous studies (7, 9, 15, 25). The RFA puncture technique for subcapsular tumors should aim to avoid the increased risk of bleeding and seeding caused by direct puncture and minimize any damage to the tumor capsule exposed on the liver surface to reduce the risk of tumor seeding (7). For RFA treatment of high-risk CLM locations, a variety of auxiliary technologies should be used to create an operating environment that can apply sufficient energy.

Artificial ascites-assisted RFA treatment for subcapsular tumors is considered safe and effective as it improves tumor visibility and obtains better electrode access, as well as helps to form a space between the liver capsule and the skin or diaphragm to avoid burns (26, 27). In addition, selecting percutaneous RFA, RFA with open surgery, laparoscopic RFA, or a combination of multiple modes according to the tumor size and location can overcome the technical difficulty of ablating subcapsular tumors and produce good ablation effects in clinical practice (28). The no-touch wedge ablation technique adopted by Patel et al. (29) for exophytic and border-deforming tumors can achieve sufficient ablation while reducing the risk of seeding or bleeding caused by tumor rupture, but its application conditions are limited. Furthermore, multimodal imaging methods are used to jointly guide the RFA procedure (CT, ultrasound, or MR) through its complementarity to improve the visibility of the lesion. Assessing the tumor immediately after ablation can clarify the coverage of the tumor through the ablation defect area, and it is also important for the rapid detection and treatment of complications. When ablating a large tumor (≥20 mm), the coverage of the tumor by the sub-electrode should be thoroughly evaluated by imaging and visualization methods to ensure that a sufficient ablation margin is obtained. Therefore, the results of efficacy and safety in subcapsular tumors, which are not statistically different from those in non-subcapsular tumors, are inseparable from the optimization of auxiliary technologies such as guidance methods (ultrasound, CT), device technology, physician experience, and auxiliary techniques (such as artificial ascites infusion and hydrodissection) (30, 31).

This study has several limitations. This was a retrospective study conducted in a single medical center, and the relevant results and conclusions need to be demonstrated in other medical institutions using a larger sample size. Second, 85.9% (171/199) of the patients in this study were followed-up in the first 36 months, and a longer survival assessment needs to be further carried out. Furthermore, the follow-up treatment of the study patients’ RFA is conducive to the control of local tumors and the prolongation of survival, such as stereotactic body radiotherapy, transcatheter arterial chemoembolization, chemotherapy, and seed implantation. The observed OS or LTPFS should therefore not be attributed only to RFA.

In conclusion, although it is difficult to achieve sufficient ablative margins in subcapsular CLMs, it is still possible to safely obtain good technical effectiveness and local tumor control through appropriate image guidance and assistive techniques. The sufficient ablative margin and technical effectiveness are protective factors for LTP, and tumor size (≥20 mm) is a risk factor for LTP.

## Data Availability Statement

The original contributions presented in the study are included in the article/supplementary material. Further inquiries can be directed to the corresponding authors.

## Ethics Statement

The studies involving human participants were reviewed and approved by The Ethical Review Committee of Sir Run Run Shaw Hospital, Zhejiang University School of Medicine. The patients/participants provided their written informed consent to participate in this study. Written informed consent was obtained from the individual(s) for the publication of any potentially identifiable images or data included in this article.

## Author Contributions

HF, XWa, JQ, WL, SX, XWu, JX, and YZ organized the database. HF performed the statistical analysis. HF and XY wrote the first draft of the manuscript. All authors contributed to the article and approved the submitted version.

## Funding

This work was supported by the National Natural Science Foundation of China (81871403 to JS), the Key Research and Development Program of Zhejiang Province (2019C03014 to JS), and the Zhejiang Provincial Natural Science Foundation of China (LY21H160041 to YZ).

## Conflict of Interest

The authors declare that the research was conducted in the absence of any commercial or financial relationships that could be construed as a potential conflict of interest.
